# Considerations for Advancing Parkinson’s Disease Research in Middle East, North Africa, and South Asia

**DOI:** 10.3389/ijph.2025.1608830

**Published:** 2025-07-25

**Authors:** Halder J. Abozait, Haseeba Khalid

**Affiliations:** ^1^Department of Medicine, College of Medicine, University of Duhok, Duhok, Iraq; ^2^ Department of Internal Medicine, Akhtar Saeed Medical and Dental College, Lahore, Pakistan

**Keywords:** Parkinson’s Disease (PD), research development, Middle East, North Africa, South Asia

To the editor,

We read with great interest the article by Khalil et al. titled “Parkinson’s Disease Database in the Middle East, North Africa, and South Asia Countries,” which details the establishment of a multicenter Parkinson’s disease (PD) database across the Middle East, North Africa, and South Asia (MENASA) [1]. This initiative represents an essential milestone in addressing the lack of region-specific PD data, and we commend the authors for establishing such an extensive and collaborative endeavor.

That said, we would like to offer four considerations that may strengthen the long-term impact and scientific utility of this project:

1. Selection bias and representativeness: while the inclusion of 20 centers across nine countries is laudable, the study relies on voluntary participation from institutions with the capacity to collect data and secure IRB approval. This may inadvertently favor tertiary centers in urban regions and neglect rural or under-resourced settings where PD diagnosis and care are more fragmented. To improve representativeness, we suggest incorporating community-based or primary healthcare data sources in future phases, or at least comparing participating vs. non-participating regions to assess coverage gaps [2].
2. Lack of longitudinal design: the current database design appears to be cross-sectional in nature. However, PD is a progressive, chronic condition where outcomes, including symptom progression, treatment response, and caregiver burden, evolve over time. A longitudinal follow-up structure, even with minimal time points, would add considerable value by enabling trajectory modeling, health economics studies, and survival analyses. We encourage the authors to consider embedding follow-up assessments in the protocol and leveraging electronic health records where feasible [3].
3. Patient and caregiver engagement: while the study commendably includes patient-perceived health status, there is limited discussion of how patients or caregivers were involved in the development of the questionnaire or governance of the consortium. Including patient representatives in design and review stages could enhance cultural appropriateness, increase trust and participation, and ensure the priorities of people living with PD are reflected in the research [4].
4. Environmental exposure assessment: the planned exploration of exposures like pesticide use is particularly valuable. Yet, pesticide types and usage patterns are likely to vary significantly between rural and urban, and within agricultural versus industrial regions. Incorporating geospatial mapping or granular exposure categories (e.g., type, duration, intensity) would significantly strengthen the ability to link environmental risk to clinical phenotypes [5].

Despite these limitations, the CGD-PD database lays the groundwork for transformative regional research. With refinement, it can serve not only as a surveillance tool but as a launchpad for interventional studies, health policy development, and equitable care models tailored to the MENASA region’s unique demographic and environmental context.
